# Frömmigkeit, Fashion und Business: Positionen ethisch/ästhetischer Weiblichkeit in muslimischen Lifestyle-Vlogs

**DOI:** 10.1007/s41682-021-00068-y

**Published:** 2021-09-03

**Authors:** Gabriel Malli

**Affiliations:** 1 Internationales Graduiertenkolleg Resonante Weltbeziehungen in sozioreligiösen Praktiken in Antike und Gegenwart, Universität Graz, Graz, Österreich; 2 Internationales Graduiertenkolleg Resonante Weltbeziehungen in sozioreligiösen Praktiken in Antike und Gegenwart, Max-Weber-Kolleg Erfurt, Erfurt, Deutschland

**Keywords:** Subjekt, Subjektpositionen, Diskursanalyse, Islam, Geschlecht, YouTube, Subjectivity, Discourse analysis, Islam, Gender, YouTube

## Abstract

Der Artikel präsentiert empirische Befunde zur Konstruktion muslimischer Weiblichkeits-Modelle in deutschsprachigen Lifestyle-Vlogs auf YouTube, in denen gläubige muslimische Frauen als Influencerinnen auftreten und ihre Alltagsroutinen und -praktiken in zumeist idealisierter Weise und unter Bezugnahme auf religiöses Wissen inszenieren. Ausgehend von poststrukturalistischen Theorieansätzen möchte ich diese Modelle als Subjektpositionen eines Diskurses muslimischer Weiblichkeit begreifen, der bestimmte Modi des Selbstverstehens und der Selbstführung sowie damit verknüpfte Affizierungsweisen nahelegt. Im Anschluss an eine kurze Diskussion islamischer und säkularer Weiblichkeitsdiskurse, in deren Schnittpunkt mein Material zu verorten ist, zeige ich anhand zweier deutschsprachiger Lifestyle-Kanäle, deren Videos ich mithilfe diskursanalytischer Verfahren untersucht habe, wie Produzentinnen ein hybrides Modell muslimischer Subjektivität hervorbringen, das bestimmte Entwürfe eines gelingenden muslimischen Lebens im westlichen Kontext enthält. Sie versuchen, alltägliche Widersprüche zwischen westlich markierter Konsum- und Populärkultur einerseits und muslimischer Religiosität andererseits auf positiv affizierende Weise aufzulösen, wobei eine Subjektposition ethisch/ästhetischer muslimischer Weiblichkeit entsteht, in der sich Ideale modischer Expressivität mit religiösen Selbstkonzeptionen und (neo-)liberalen Diskursen von Autonomie und Selbst-Unternehmertum verschränken.

**Zusatzmaterial online:**

Zusätzliche Informationen sind in der Online-Version dieses Artikels (10.1007/s41682-021-00068-y) enthalten.

## Abstract (en)

The article discusses empirical findings on the construction of models of Muslim femininity in German-speaking lifestyle vlogs on YouTube, in which faithful Muslim women appear as influencers and stage their everyday routines and practices in a mostly idealized way and with references to religious knowledge. Applying a post-structuralist theoretical framework, I consider those models as subject positions of a specific discourse of Muslim femininity promoting certain modes of self-understanding, self-conduct and related ways of affection. After a brief discussion of Muslim and secular discourses of femininity in which my data can be located, I present results from a discourse analysis of videos from two German-speaking channels: I suggest that the producers constitute a hybrid model of Muslim subjectivity which encompasses templates of a successful Muslim life in a secular society. In this context, producers seek to dissolve everyday contradictions and tensions between Western consumer and popular culture on the one, and Muslim religiosity on the other hand. By doing so, they produce a position of ethical/aesthetical Muslim femininity that brings together ideals of fashionable expressivity with religious self-conceptions and (neo-)liberal discourses of female autonomy and self-entrepreneurship.

## Einleitung: Vlogging Gender

Die Figur der muslimischen Frau ist im westlichen Kontext ein diskursives Kampffeld: Regelmäßig werden ihre vermeintlichen Lebensumstände und Praktiken in Debatten thematisiert, an denen nicht zuletzt akademische Akteure partizipieren. So entstand in den letzten Jahren im deutschsprachigen Raum eine große Anzahl sozial- und kulturwissenschaftlicher Publikationen, die sich empirisch oder theoretisch mit Weiblichkeit im Islam befassen. Wie Amir-Moazami (2018, S. 9 f) kritisch anmerkt, fügen sich einige der Arbeiten in Wissensordnungen, die den Islam als ein nicht-europäisches „Anderes“ hervorbringen und politische Interventionen gegen bestimmte Formen islamischen Lebens anregen. Andere, an diskurstheoretische oder postkoloniale Zugänge anschließende Arbeiten rücken hingegen die diskursive und performative Konstitution muslimischer Weiblichkeiten in den Vordergrund: Einerseits konzentrieren sich Autor_innen auf den Meta-Diskurs *über* den Islam und machtvolle diskursive Zuschreibungen an muslimische Frauen durch nicht-muslimische Sprecher_innen (Oestreich 2004; Jäger und Jäger 2007; Shooman 2014; Korteweg und Yurdakul 2016; Hark und Villa 2017); andererseits liegt der Fokus darauf, wie muslimische Frauen selbst ihre Geschlechterpraxis im Spannungsfeld religiöser und säkularer Weiblichkeitsnormen verhandeln (Klinkhammer 2000; Nökel 2002; Amir-Moazami 2007; Şahin 2014).

Der vorliegende Artikel knüpft an den zweiten Strang an und beschäftigt sich mit der Konstruktion muslimischer Weiblichkeit durch muslimische Frauen in so genannten Vlogs auf der Video-Plattform YouTube. Bei Vlogs handelt es sich um üblicherweise amateurhaft produzierte Videobeiträge, in denen Produzent_innen in direkten Kontakt mit dem Publikum treten, es persönlich ansprechen, von alltäglichen Erfahrungen berichten oder ihre Alltagspraxis dokumentieren (vgl. Werner 2012 für einen Überblick zum Phänomen). Dabei lassen sich zahlreiche Genres differenzieren (etwa Lifestyle oder Fashion & Beauty, vgl. Geimer und Burghardt 2017, S. 28), wobei Produzent_innen angesichts der immensen Popularität von YouTube vor allem unter Jugendlichen^1^ einen Prominentenstatus erreichen und zu Rollenmodellen werden können (vgl. Döring 2014). Die Plattform kann folglich als wichtige Sphäre gegenwärtiger Populär- und Jugendkultur betrachtet werden, in der sich aktive Prozesse der Produktion, Rezeption und Weiterverarbeitung kultureller Wissens- und Vermittlungsformen nachvollziehen lassen (vgl. Knoblauch 2009, S. 238). Wird religiöses Wissen in populärkulturellen Medien und Formaten wie Vlogs verhandelt, lässt sich im Anschluss an Forbes (2005, S. 15ff) vielfach eine Art „Dialog“ attestieren: Religiöse und nicht-religiöse Referenzsysteme werden simultan aktiviert, konkurrieren miteinander oder verschmelzen zu neuen kulturellen Formationen.

Ausgehend davon untersucht der Artikel, welche Modelle „kompetenter“ muslimischer Weiblichkeit in den Vlogs hervorgebracht werden, mit welchen Eigenschaften, Fähigkeiten, Praktiken und affektiven Stilen diese verknüpft werden und in welchen Verhältnissen sie zu breiteren religiösen und säkularen Geschlechterdiskursen stehen. Dafür beziehe ich mich auf poststrukturalistische Theorieansätze, die ich im zweiten Abschnitt umreiße: Die genannten Modelle sollen als diskursiv erzeugte Subjektpositionen begriffen werden, die bestimmte Weisen des Selbstverstehens, der Selbstführung und des Affiziert-Werdens nahelegen. Spezifika religiöser Subjektpositionen und die Rolle neuer sozialer Medien in ihrer Vermittlung wird im dritten Abschnitt umrissen. Im vierten Abschnitt skizziere ich in Kürze die Felder islamischer und säkularer Weiblichkeitsdiskurse sowie „westlicher“ Diskurse zum Islam, in denen die analysierten Videos zu verorten sind. Im Anschluss an einen kurzen Überblick zu methodischen Überlegungen in Abschnitt 5 präsentiere ich im sechsten Abschnitt Befunde aus einer vergleichenden Analyse zweier deutschsprachiger YouTube-Kanäle, in denen muslimische Frauen Vlogs veröffentlichen. Wie ich vorschlagen möchte, kann die von ihnen hervorgebrachte Subjektposition als Hybridmodell verstanden werden, das versucht, widersprüchliche Anforderungen an muslimische Weiblichkeit in einer positiv affizierenden Weise aufzulösen.

## Geschlechtliche Subjektpositionen und ihre affektive Animation: Theoretische Grundlegungen

Ist in neueren (kultur-)soziologischen Arbeiten vom Subjekt die Rede, beziehen sich Autor_innen vielfach auf eine post-strukturalistische Theorietradition, die auf Foucault zurückgeht und die historische, soziale und kulturelle Bedingtheit von Selbst-Verhältnissen in den Vordergrund rückt (vgl. Reckwitz 2008, S. 9 f): Ein Individuum wird demnach zum Subjekt, indem es praktisch einen soziohistorisch verorteten Modus des Selbstverstehens und der körperlichen Selbstführung annimmt, durch den es für sich und andere verstehbar wird. Ein solcher Modus steht wiederum mit machtdurchdrungenen diskursiven Wissensordnungen in Verbindung^2^: Subjekte müssen insofern als *Subjekte eines Diskurses* begriffen werden, als dass ihre gesamte soziale Existenz notwendigerweise von Normen und Kategorien strukturiert wird, die sie nicht selbst gewählt haben (vgl. Butler 2019, S. 20). Dabei stellt Geschlechtlichkeit eine zentrale diskursive Kategorie dar: Sich selbst als Subjekt eines Geschlechterdiskurses zu verstehen und dementsprechend zu führen, stellt eine ubiquitäre Anforderung für Individuen dar. Die dominante Geschlechterordnung moderner patriarchaler Gesellschaften ist eine binäre, die hierarchisierende Differenzen zwischen den exklusiven Kategorien männlich und weiblich errichtet (vgl. Butler 1999).

Ein empirischer Zugriff auf vergeschlechtlichte Subjektnormen kann über die Analyse von Subjektpositionen erfolgen, die in Diskursen als „sozial bewohnbare Zonen“ (Villa 2013, S. 66) für Adressat_innen hervorgebracht werden: Es handelt sich dabei um normativ wirksame Modelle und Anforderungsprofile für „kompetente“ (oder „inkompetente“) Subjektivität (vgl. Reckwitz 2008, S. 26 f), die Einzelnen Ressourcen bieten, sich als Subjekte einer bestimmten Geschlechterordnung anzuerkennen. So zirkulieren etwa essentialisierende Positionen einer „wahren“ (oder auch „falschen“) Weiblichkeit bzw. Männlichkeit inklusive angelagerter Modellpraktiken in diversen gesellschaftlichen Feldern (vgl. Villa 2011, S. 181). Einzelne stehen folglich unter fortwährendem sozialen Druck, sich gegenüber diesen Subjektivierungsformen zu positionieren, wobei sich die Reproduktion – oder Subversion – von Geschlechternormen in einer ständigen Abfolge konkreter, vielfach routinisierter performativer Akte vollzieht (vgl. Butler 1999, S. 173). Hierbei tritt die aktive Komponente von Subjektivierung hervor: So kann die Aneignung, Verwerfung oder Subversion von Subjektnormen als lustvolle oder ermächtigende Prozedur aufgefasst werden, in der Individuen „aus ihrem Leben ein Werk zu machen suchen, das gewisse ästhetische Werte trägt“ (Foucault 2020, S. 18), und Identitäten aufbauen, indem sie sich positiv an eine Subjektposition „verhaften“ (vgl. Butler 2019, S. 9ff).

Insbesondere in der Postmoderne lässt sich eine verstärkte Konkurrenz von Subjektpositionen und ihren Anforderungsprofilen feststellen (vgl. Reckwitz 2020, S. 605), die in diskursiven Deutungskämpfen an die Oberfläche treten: Das Individuum ist gezwungen, sich in einem Feld widersprüchlicher Subjektmodelle zu positionieren, was zu intrapersonalen Spannungen und Identitätskonflikten führen kann. Dabei wird auch deutlich, dass Subjektivierung nie als abschließbarer Prozess zu verstehen ist und die an den Einzelnen herangetragenen Erwartungen nie vollständig erfüllt werden können (vgl. Bröckling 2007, S. 28): Das Subjekt ist weder stabil noch fixiert, vielmehr befindet es sich in einen fortlaufenden Orientierungsprozess, in dem es sich in den als erstrebenswert aufgefassten Kompetenzen „trainiert“ (vgl. Reckwitz 2020, S. 29).

Weiter ist anzumerken, dass Subjektpositionen nicht nur durch Selbstverständnis und Selbstpraxis, sondern auch durch Affektstrukturen^3^, emotionale Haltungen sowie bestimmte Weisen des Begehrens gekennzeichnet sind (vgl. Reckwitz 2008, S. 137), die in männlich oder weiblich kodierten Varianten auftreten. Ein „richtiges“ Fühlen gemäß einem „emotionalen Kanon“ (Wetherell et al. 2015) und eine als angemessen wahrgenommene affektive Praxis in Bezug auf spezifische Objekte, Akteure oder Konzepte sind zentrale Bestandteile kompetenter Subjektivität. In diskursiven Formationen werden „bestimmte Körper, Objekte und Sprechakte mit bestimmten Arten, affiziert zu sein“ (von Scheve 2017), in Verbindung gesetzt, wodurch sie „animiert“ werden und gewissermaßen „zum Leben erwachen“ (vgl. Ahmed 2004, S. 118). Eine solche Animation von Subjektpositionen durch die Zuschreibung von Emotionen ermöglicht es Einzelnen, mit ihnen in Beziehung zu treten: Werden zugeschriebene Emotionen tatsächlich gefühlt, gewinnt das Modell an Plausibilität, und Voraussetzungen für eine längerfristige Verhaftung entstehen (vgl. Berg et al. 2019, S. 53).

Während ein empirischer Zugang zu „tatsächlichen“ Affekten über diskursanalytische Verfahren problematisch erscheint, lassen sich in Diskursen „affektive Kopplungen“ (Berg et al. 2019, S. 49) rekonstruieren, durch die einzelne „Diskurskörper“ zueinander in Verbindung gesetzt werden. Subjektpositionen können als ein solcher Körper verstanden werden, der sich in Diskursen affektiv an disparate Objekte, Ideen oder Personen koppelt: So ist beispielsweise das kompetente weibliche Subjekt eines „spätbürgerlichen“ Geschlechterdiskurses durch heteroromantische Liebe zum Ehemann, eine emphatische und intime Beziehung zum Kind oder eine positive Verhaftung an das Häusliche, das als Ort besonderer emotionaler Dichte erscheint, gekennzeichnet (vgl. Reckwitz 2020, S. 265ff).

## Weibliche Moralität im religiösen Cyber-Diskurs: Theoretische Erweiterungen

### Religiöse Subjektivität

Ausgehend von den bisherigen Überlegungen lässt sich religiöse Subjektivität als Weise des Selbstverstehens und der Selbstführung begreifen, die maßgeblich in glaubensbasierten Diskursen begründet ist. Zentral hierfür sind Kategorien und Techniken, die auf eine Zurichtung des Selbst im Sinne einer religiösen Moral abzielen und dabei Körper‑, Interaktions- oder Ritual-Praktiken adressieren. Im Anschluss an das Spätwerk Foucaults möchte ich hier auf die ethische Dimension religiöser Subjektivierungsformen fokussieren: Zentral hierfür ist die als normatives Ideal hervorgebrachte „Art der Beziehung, die man zu sich selbst haben sollte, [den] Selbstbezug, […] der bestimmt, wie das Individuum sich als Moralsubjekt seiner eigenen Handlungen konstituieren soll“ (Foucault 1994, S. 275). In einer Analyse ethischer Subjektpositionen sind weiter vier Aspekte zu berücksichtigen (vgl. Foucault 2020, S. 37–40): Erstens ist nach der *ethischen Substanz* zu fragen, womit Foucault den Aspekt des Selbst meint, der moralisch geführt werden soll (etwa die Gefühle oder der Körper). Als zweiten Aspekt nennt er die spezifische *Unterwerfungsweise* eines moralisch-religiösen Diskurses, wofür die Instanzen zu rekonstruieren sind, deren Autorität den Einzelnen zur Anerkennung seiner moralischen Verpflichtungen veranlasst (etwa ein göttlicher Wille oder eine kosmologische Ordnung) (vgl. Foucault 1994, S. 276). Drittens geht es Foucault um *Techniken der Selbstbearbeitung* (etwa Gebete, Übungen oder Verhaltenscodes), die zur Realisierung ethischer Subjektivität anzuwenden sind; viertens um den *Telos*: die als begehrenswert hervorgebrachte Art moralischen Seins, an die eine Bindung erzeugt werden soll (vgl. ebd. S. 277). Ethische gehen dabei mit ästhetischen Werten einher, zumal Subjektivierungsprozesse entlang moralischer Maßgaben zwangsläufig Momente ästhetischer Stilisierung aufweisen (vgl. Elberfeld und Otto 2009, S. 21 f).

Moralisch-religiöse Subjektpositionen reflektieren vielfach religiös begründete Geschlechterdiskurse, die vorgeben, „was Männer und Frauen kennzeichnet bzw. wie sie zu sein haben, [woraus] folgt, was man als Angehörige eines Geschlechts zu tun und was dagegen zu lassen hat“ (Benthaus-Apel et al. 2017, S. 9). Basierend darauf ist ethisch kompetente Weiblichkeit an andere (und vielfach restriktivere) Erwartungshaltungen gekoppelt als ihr männliches Pendant, wobei die „symbolische Verkörperung der ‚Reinheit‘ der Gruppe [in] Differenz gegenüber der ‚ungläubigen‘ Umwelt“ (Wohlrab-Sahr und Rosenstock 2000, S. 296) eine häufig weiblichen Subjekten zugeschriebene Aufgabe darstellt.

In säkular verfassten und religiös pluralen Gesellschaften werden moralisch-religiöse Wissensformen und Subjektpositionen von unterschiedlichen Sprecher_innen laufend herausgefordert: Zum einen finden innerhalb religiöser Felder Deutungskämpfe statt, die sich um Fragen nach der richtigen religiösen Praxis formieren und die spezifischen religiösen Interessen gesellschaftlicher Gruppen reflektieren (vgl. Bourdieu 2009, S. 57ff); zum anderen kommt es zu Anfechtungen durch säkulare Sprecher_innen, die die Legitimität glaubensbasierter Kategorien als Ressourcen für Subjektivierungsprozesse generell in Frage stellen (vgl. Asad 2003). Zudem wird das Phänomen einer Entgrenzung des Religiösen beschrieben (vgl. Knoblauch 2009, S. 198ff): So diffundieren religiös geprägte Symbole und Diskurse etwa zunehmend in das Feld der Populärkultur, wo sie kulturell neu konfiguriert werden. Häufiges Resultat sind hybride Subjektformen und Identitätsentwürfe, in denen sich moralisch-religiöse mit anderen sozialen Referenzsystemen (etwa politischen, kulturellen oder ökonomischen) überlappen (vgl. Reckwitz 2020, S. 624).

### Religiöse Subjekte und digitale Medien

Ausgehend von Befunden einer zunehmenden „Mediatisierung von Subjektivierungsprozessen“ (Geimer und Burghardt 2019) möchte ich neue soziale Online-Medien als „Medien-Dispositiv“ begreifen (vgl. Hickethier 2010, S. 197 f), das spezifische Beziehungsnetzwerke zwischen Betreiber_innen, Nutzer_innen und Inhalten etabliert. Innerhalb eines solchen Netzes zirkulieren religiöse Subjektpositionen und damit verknüpfte Affekte, wobei eine Stabilisierung ebenso wie eine Herausforderung von Normen möglich ist (vgl. Campbell 2010). Abermals ist die privilegierte Rolle der Kategorie Geschlecht in Social-Media-Diskursen zu erwähnen: So lässt sich am Fall von YouTube zeigen, dass Produzent_innen vielfach Geschlechterstereotype aufgreifen, wodurch sie hegemoniale Modelle männlicher bzw. weiblicher Subjektivität reproduzieren und Geschlechterdifferenzen naturalisieren (vgl. Döring 2019, S. 4 f). Für den Fall religiöser Geschlechtermodelle wird hingegen auch das Potenzial einer Herausforderung tradierter Normen hervorgehoben (vgl. Lövheim 2013, S. 58ff).

Eine besondere Position im digitalen Diskursraum nimmt die in den 2010er-Jahren entstandene Figur der Influencer_in ein (vgl. Nymoen und Schmitt 2021): Dabei handelt es sich um Content-Produzent_innen, die aufgrund ihrer hohen Reichweite in sozialen Medien zur gezielten Werbung für zumeist kommerzielle Angebote angeworben werden oder diese eigenständig betreiben. Die Vermittlung an das Publikum erfolgt üblicherweise in Form „authentischer“ Produkt-Empfehlungen, wobei die Erzeugung affektiver Beziehungen zwischen Darsteller_innen, Publikum und den beworbenen Objekten einen zentralen Bestandteil der von Influencer_innen zu bewältigenden Arbeit darstellt (vgl. Peterson 2017, S. 8 f). Viele Produzent_innen versuchen darüber hinaus selbst, sich strategisch als potenzielle Influencer_innen in Stellung zu bringen und Aufmerksamkeit von Unternehmen zu generieren (vgl. Hearn und Schoenhoff 2016). Daraus kann die Konstitution von Subjektivierungsformen resultieren, die zu einem wesentlichen Teil über Konsumpraktiken definiert sind, vermittelt über Logiken des Gender Marketing dominante Geschlechterdiskurse aktualisieren (vgl. Krell 2009) und Schablonen legitimer geschlechtlicher (Affekt‑)Praxis konstituieren. Eine Sonderform stellt die Figur der religiösen Influencer_in dar (vgl. Beta 2019, S. 2146): Dabei handelt es sich um Produzent_innen, die kommerzielle Interessen mit religiösen Appellen an ihr Publikum kombinieren, indem sie etwa bestimmte Konsumpraktiken und -produkte als ethisch und der Realisierung moralischer Subjektivität zuträglich bewerben.

Zusammenfassend lässt sich festhalten, dass soziale Medienplattformen Zirkulationssphären für Diskurse eröffnen, in denen vergeschlechtlichte moralisch-religiöse Subjektpositionen – verstanden als Modelle richtiger (oder falscher) religiöser Weiblichkeit bzw. Männlichkeit im Verbund mit entsprechenden Modellpraktiken – performativ hervorgebracht, normativ aufgeladen und neu konfiguriert werden, wobei sie mit emotionalen Gehalten verknüpft werden. Dabei stehen religiöse Subjektformen stets in verschieden gearteten Beeinflussungs- und Konkurrenzverhältnissen mit anderen, etwa säkularen Modellen, die gegenläufige Anforderungen an „kompetente“ Geschlechtlichkeit stellen.

## Muslimische Weiblichkeit im Spannungsfeld von Geschlechterdiskursen: Ein Überblick

Im Anschluss an diese theoretische Herleitung wagt der folgende Abschnitt eine knappe Skizze säkularer und muslimischer weiblicher Subjektpositionen und bereitet so eine „Topographie“ des diskursiven Feldes auf, in dem die anschließend analysierten Fälle zu verorten sind. So handelt es sich bei den gegenständlichen Videos um Medien, die von jungen, sich als muslimisch verstehenden Frauen im Kontext nicht-muslimischer, tendenziell säkularer Dominanzgesellschaften produziert werden und ein näher zu analysierendes Modell muslimischer Weiblichkeit hervorbringen. Dabei befinden sie sich im Spannungsfeld gegenläufiger Diskurse, die um Probleme im Zusammenhang mit (islamischer) Geschlechtlichkeit zirkulieren.

Aus diesem diskursiven Feld ragt ein Komplex an säkularen Subjektpositionen hervor, die im Kontext westlicher, nicht-muslimischer Gesellschaften weitestgehend als hegemonial betrachtet werden können und in verschiedenen Kontexten an junge Frauen herangetragen werden. Zu nennen ist zunächst das im spätbürgerlichen Geschlechterdiskurs des 19. Jahrhunderts verwurzelte, biologisch begründete und in der Figur der fürsorgenden Mutter hervortretende Modell empfindsamer und kommunikativer Weiblichkeit, wobei ein dem ökonomisch tätigen Mann zu- und untergeordnetes Ehesubjekt das Ideal darstellt (vgl. Reckwitz 2020, S. 164ff; siehe auch Eckes 2010). Demgegenüber steht ein vielfach von liberal-feministischen Sprecher_innen vorgebrachtes Anforderungsset an kompetente weibliche Subjektivität, das autonomes Handeln akzentuiert und das weibliche Subjekt dann als zur Selbstverwirklichung befähigt ansieht, wenn es auf Basis eigener Präferenzen ohne Fremdzwänge freie Entscheidungen zwischen Handlungsoptionen treffen kann (vgl. Mahmood 2005, S. 12ff).

Im Zentrum eines weiteren dominanten Sets weiblicher Subjektprofile steht die „gekonnte ästhetische Stilisierung des Körpers nach außen“ (Reckwitz 2020, S. 566) durch Konsum- und Selbstsorgepraktiken, mittels derer sich Frauen als Subjekt eines Schönheitsdiskurses hervorbringen sollen. Machtvolle Ideale (etwa Schlankheit, Eleganz oder Sexyness) wirken über Selbsttechnologien (etwa Kleidungspraktiken oder Diäten, vgl. Gesing 2006) auf den weiblichen Körper ein, der Gegenstand dauerhafter Selbst- und Fremdbeurteilungen ist (vgl. Bartky 1997, S. 139 f). Auch wenn vergeschlechtlichte Normierungstechniken in verschiedenen sozialen Bereichen zur Anwendung kommen, ist an dieser Stelle die Wirkmächtigkeit populärkultureller Weiblichkeitsrepräsentationen und -performanzen zu betonen, die in besonderem Maße in der Lage sind, Aussagen eines Geschlechterdiskurses zu popularisieren (vgl. Villa et al. 2012, S. 14 f).

Diese Diskursstränge treten wiederum in der Beurteilung des Islams im deutschsprachigen Raum in Erscheinung und überschneiden sich in der Figur der verschleierten Frau: Diese wird einerseits im Rahmen einer ästhetisch-körperlichen, andererseits im Rahmen einer liberal-feministischen Weiblichkeitsnorm als defizitär wahrgenommen. Häufig sind Zuschreibungen der Unterdrückung, wobei das Kopftuch weibliche Handlungsfähigkeit und Selbstverwirklichung unterminiere^4^. Die reale Polysemie islamischer Geschlechterpraktiken wird zurückgewiesen (vgl. Hark und Villa 2017, S. 71), ein Druck zur Verschleierung durch Familie oder Community als repräsentativ für einen homogen gedachten Islam dargestellt (vgl. Amir-Moazami 2007, S. 120). Darüber hinaus werden Verschleierungspraktiken mitunter als Symbole politisch-islamischer Bewegungen interpretiert, die Prinzipien des Liberalismus, des Säkularismus oder der Geschlechtergleichheit in Frage stellen würden (vgl. Rostock und Berghahn 2009, S. 12). Daraus resultiert ein paradoxes, aber machtvolles Negativmodell einer muslimischen Frau, die einerseits zum passiven Opfer von Unterdrückung, andererseits als Trägerin eines islamistischen Symbols zur aktiven Bedrohung für „westliche“ Werte stilisiert wird (vgl. Bilge 2010, S. 15–18). Eine Positivschablone stellt hingegen die säkularisierte, „entschleierte“ muslimische Frau dar, die ihre religiöse Praxis ausschließlich im Privaten lebt und sich an einem assimilierten „Euro-Islam“ (Tibi 2009) orientiert.

In durch explizit islamische Sprecher_innen getragenen Weiblichkeitsdiskursen finden sich unterschiedliche Strategien des Umgangs mit hegemonialen säkularen Geschlechtermodellen und den daraus hergeleiteten Zuschreibungen und Anforderungen an muslimische Frauen^5^: Eine Strategie besteht in einer klaren Abgrenzung von säkularen Idealen autonomer Selbstverwirklichung oder körperlicher Ästhetisierung, wohingegen die Frage nach der richtigen Praxis explizit religiöser weiblicher Tugenden – etwa Frömmigkeit, Schamhaftigkeit, Ehefrauen- oder Mutterschaft – im Kontext moderner Gesellschaften in den Vordergrund rückt (vgl. Mahmood 2005 für eine Reihe von Fallstudien). Vor dem Hintergrund des „Islamic Revival“ zielen Positionen aus verschiedenen islamischen Kontexten auf ein ethisches Ideal ab, das vollkommene weibliche Subjektivität an eine Selbstkultivierung entlang strikter moralischer Codes koppelt, die zugleich als ästhetische Werte hervorgebracht werden. Kompetente Weiblichkeit ist hier durch die Unterwerfung unter göttlichen Willen gekennzeichnet und realisiert sich in performativen Akten wie „bedeckenden“ Bekleidungspraktiken (allen voran dem Tragen des Kopftuchs) oder dem „schamhaften“ Umgang mit nicht-verwandten Männern (vgl. ebd. S. 23–27; Abu-Lughod 2005). Davon abzugrenzen sind verschiedene ethnisch-kulturelle Weiblichkeitsideale, die vielfach in islamisch geprägten, postmigrantischen Milieus anzutreffen sind und sich ebenfalls in Opposition zu säkularen Normen befinden, allerdings weniger von theologischen Konzepten als von diskursiven Traditionen der jeweiligen Herkunftsländer geprägt sind (vgl. Salvatore und Amir-Moazami 2002, S. 324 f).

Dem gegenüber stehen Modelle einer Verschränkung islamischer und säkularer Weiblichkeitspositionen, deren Vermittlung nicht zuletzt über populärkulturelle Medien erfolgt: Genannt werden können hier „moderne“ Formen islamischer Weiblichkeit, für die religiöse Konzepte eine flexibel einsetzbare Ressource zur Alltagsbewältigung oder Elemente „privater“ spiritueller Praxis (vgl. Moors und Tarlo 2013, S. 19) sind. In der Literatur werden zudem die Phänomene eines „Neo-Islams“ (Nökel 2002) oder „Pop-Islams“ (Gerlach 2010) beschrieben: Dabei handelt es sich um islamische Lebensformen in westlichen Kontexten, in denen sich ein akzentuiert religiöses Selbstverständnis, eine Abgrenzung vom kulturellen Islam der Elterngeneration, das Streben nach beruflicher und sozialer Partizipation in der Mehrheitsgesellschaft sowie eine Offenheit gegenüber bestimmten Formen westlicher Jugendkultur überkreuzen (vgl. auch Herding 2014). Ebenso lassen sich Tendenzen einer verstärkten Selbstästhetisierung über explizit modische islamische Kleidungspraktiken erkennen, die mit dem Aufschwung einer Industrie für muslimisch-religiöse Mode einhergeht (vgl. Moors 2013, S. 19 f) und in neuen sozialen Medien befördert wird. Dort markieren die transnational verwendeten Selbstbezeichnungen „Hijabi“ und „Hijabista“ (Neologismus aus Hijabi und Fashionista) eine Form muslimischer Subjektivität, die religiöse Identität und modische Weiblichkeit verknüpfen (vgl. Kavakci und Kraeplin 2017; Braun 2019, S. 89).

Muslimische Frauen im deutschsprachigen Raum befinden sich folglich in einer Situation, in der sie laufend als Subjekte widersprüchlicher Weiblichkeitsdiskurse angerufen werden (vgl. Amir-Moazami 2018, S. 11). Göle (2013, S. 6) beschreibt einen daraus resultierenden „Status konstanter Reflexivität“ als „Pendelbewegung“ zwischen konkurrierenden religiösen und säkularen Ansprüchen, die Spannungen auf intrapersonaler Ebene erzeugt, fortwährende Anpassungs- und Aushandlungsprozesse erfordert und mitunter hybride Identitäten, verstanden als ein „Verschmelzen“ verschiedener identitärer Referenzsysteme im Selbst (vgl. Foroutan 2013, S. 89f), hervorbringt. Ausgehend von diesen Befunden soll nun im Hinblick auf das Analysematerial die Frage adressiert werden, welche Konfigurationen islamischer Weiblichkeit in den Videos sichtbar werden, welche Diskurse sich in ihnen verschränken, und wie sie an Spannungsverhältnisse appellieren.

## Analyse von Subjektpositionen auf YouTube: Die Empirische Strategie

Nachdem ich im dritten Abschnitt das theoretische Argument entwickelt habe, dass Social-Media-Plattformen Räume zur Verhandlung moralisch-religiöser und vergeschlechtlichter Subjektpositionen eröffnen, wurden im vierten Teil Ausschnitte des diskursiven Spannungsfeldes skizziert, in dem sich Subjektivierungsprozesse muslimischer Frauen in säkularen Gesellschaften vollziehen. In Folge werde ich einen hybriden Modus muslimisch-weiblicher Subjektivität, der in YouTube-Videos performativ hervorgebracht und affektiv „verkoppelt“ wird, genauer analysieren. Der folgende Abschnitt umreißt in Kürze meine empirische Vorgehensweise.

### Schritt 1: Die Identifikation einer diskursiven Position

Im Zuge digitaler Feldforschung auf YouTube konnte zunächst ein Cluster nutzer_innengenerierter Kanäle und Videos identifiziert werden, in denen muslimische Frauen und (Ehe‑)Paare ihren Alltag unter Rückgriff auf Elemente des Vlogging-Genres dokumentieren und sich ähnlicher ästhetischer Formen und Vermittlungsweisen bedienen. Dabei wurde ich auf zehn deutschsprachige YouTube-Kanäle aufmerksam, die trotz unterschiedlicher thematischer Schwerpunktsetzungen (etwa Familie, Partnerschaft, Mode, Styling oder Shopping) unter das gemeinsame Label des muslimischen Lifestyle-Kanals gefasst werden können und zur Konstruktion einer religiös basierten Konsument_innen-Kultur beitragen (vgl. Lewis 2010, S. 68 f). Zentral ist die Präsenz islamischer Symbole und Referenzen in den Produktionen: Die Sprecherinnen tragen Kopftuch, streuen religiöse Formeln in ihr Sprechen ein oder beziehen sich auf islamische Konzepte, Rituale oder Feiern. Ein solches Feld der medialen Performance islamischen Lifestyles lässt sich freilich nicht auf YouTube eingrenzen. Insbesondere auf der Fotoplattform Instagram werden ähnliche Themen und Motive – in vorwiegend unbewegter Form – inszeniert (vgl. Baulch und Pramiyanti 2018), wobei auch die meisten der von mir untersuchten YouTuber_innen zugleich Instagram-Kanäle betreiben, auf die sie in ihren Videos verweisen. Ebenso handelt es sich um keinen rein deutschsprachigen Diskurs, so werden ähnliche Kanäle im türkischen, arabischen und angelsächsischen Raum betrieben (vgl. Kavakci und Kraeplin 2017; Peterson 2017).

### Schritt 2: Auswahl der Kanäle und Videos

Die folgende Analyse berücksichtigt Videos zweier deutschsprachiger Kanäle, nämlich „Eurasian Muslima“ (mittlerweile unter dem Titel „Nataya’s Life“ geführt) und „kubraxdeniz“. Diese Auswahl erfolgte einerseits aufgrund ihrer relativ hohen Reichweite, andererseits unter Berücksichtigung ihrer inhaltlichen Diversifizierung – so sind sie nicht auf einzelne Aspekte muslimischen „Lifestyles“ eingeschränkt, sondern behandeln jeweils ein breiteres Spektrum an Themen, wobei Familienleben, Partnerschaft, Mode, Styling, Hausarbeit, Rezepte, Shopping, Freizeitgestaltung und religiöse Praxis wiederkehrende Elemente darstellen. Damit weisen sie das Potenzial auf, ein umfassenderes Bild von Subjektpositionen eines westlich-muslimischen Lifestyle-Diskurses zu zeichnen als etwa Kanäle, die sich lediglich auf eines dieser Themen beschränken. In meinen Materialkorpus wurden letztendlich 46 Videos aus dem Zeitraum zwischen Anfang Juli 2018 und Ende Juni 2019 einbezogen. Diese zeitliche Eingrenzung stellt letztlich eine pragmatische Entscheidung dar, wobei anzumerken ist, dass im Zuge der Video-Analysen eine „theoretische Sättigung“ im Sinne der Grounded Theory (vgl. Glaser und Strauss 2006, S. 61) eintrat, die Beiziehung zusätzlichen Materials also keine zusätzlichen Ergebnisse mehr hervorbrachte. (Tab. 1).

**Table 1:** 

Name	Statistik
EM … Eurasian Muslima [seit 2020 „Nataya’s Life“]	Gegründet Januar 2014
	7023 Abonnent_innen (07/2018) – 21.993 Abonnent_innen (07/2019)
	370.649 Video-Aufrufe (07/2018) – 1.742.984 Video-Aufrufe (07/2019)
	Veröffentlichung von 26 Videos im Erhebungszeitraum
KXD… kubraxdeniz	Gegründet Oktober 2018
	23.197 Abonnent_innen (07/2019)
	1.919.554 Video-Aufrufe (07/2019)
	Veröffentlichung von 30 Videos im Erhebungszeitraum

### Schritt 3: Datenanalyse

Im Anschluss an die in Abschnitt 2 entfalteten theoretischen Argumente zielt meine Analyse darauf ab, sowohl sprachliche Aussagen als auch affektive „Affordanzen“ der einzelnen Videos zu rekonstruieren, in denen sich bestimmte Subjektpositionen formieren. Ausgehend vom Befund neuerer diskursanalytischer Ansätze, wonach sich Diskurse zwar über verschiedene semiotische Modi entfalten können, Sprache dabei aber eine privilegierte Position einnimt (vgl. Keller 2016, S. 79), habe ich die Videos sowohl auf der Ebene des gesprochenen Textes als auch auf der der Visualitäten zunächst mit einem an das „offene Kodieren“ der Grounded Theory (vgl. Corbin und Strauss 2015, S. 222ff) angelehnten Kodierverfahren untersucht und dabei Kategorien entwickelt, die sich einerseits auf Kapazitäten und Qualitäten des in den Videos konstruierten Idealsubjekts, andererseits auf die vorgeschlagenen Praktiken zur Realisierung eines solchen Selbstverhältnisses beziehen.

In einem zweiten Analysedurchgang habe ich unter Anwendung des an Sara Ahmed anschließenden Analyseverfahrens des „Reading for Affect“ (Berg et al. 2019) nachgezeichnet, wie Subjektpositionen unter Anwendung bestimmter emotionsbezogener Worte und Ausdrucksweisen animiert werden und sich mit dargestellten Gegenständen, Praktiken oder Konzepten affektiv „verkoppeln“ (vgl. ebd. S. 52). Ich möchte darauf hinweisen, dass ich im Rahmen der Analyse auf der Ebene des (multimodal gedachten) Medientextes bleibe: Von vorrangigem Interesse sind nicht etwa Prozesse medialer Produktion oder Aneignungs- und Rezeptionspraktiken, sondern eine diskursive Position islamischer Weiblichkeit mit Appellcharakter samt angelagerter Modellpraktiken und Affektstrukturen, die über die Medien vermittelt werden. Dafür möchte ich die zwei betrachteten Kanäle in Form einer Fallanalyse einzeln besprechen und die in ihnen ineinanderfließenden Diskurse isolieren, um im Anschluss daran die ihnen gemeinsamen Elemente, die als charakteristisch für das hervorgebrachte Subjektmodell gedacht werden können, herauszuarbeiten.

## Muslimischer Lifestyle zwischen Ethik, Ästhetik, Konsum und Kommerz: Rekonstruktion einer „gevloggten“ Subjektposition

**Figure 1:**
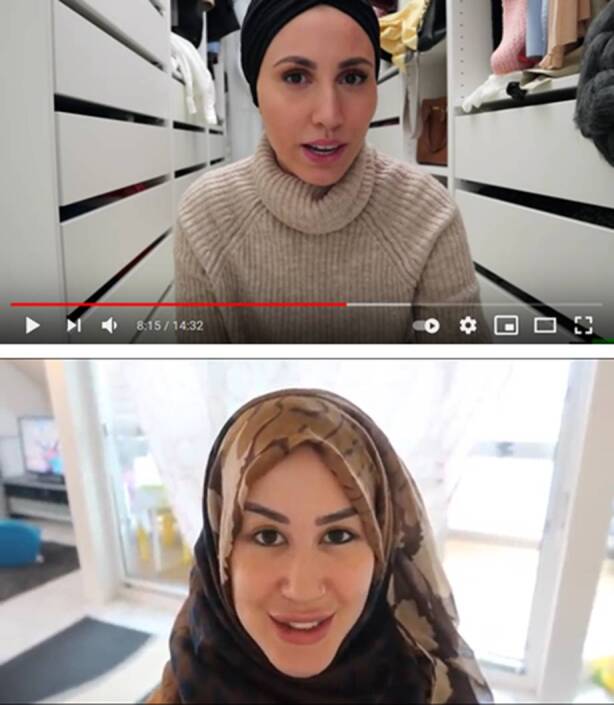

Vorwegzunehmen ist, dass die Videos grundsätzlich zwei Möglichkeiten einer subjekttheoretischen Analyse eröffnen: Einerseits können sie als Subjektivierungspraktiken ihrer Produzent_innen gelesen werden, durch die sich diese selbst als muslimische Subjekte hervorbringen (vgl. dazu Geimer und Burghardt 2019). Andererseits tragen sie zur (Re‑)Produktion einer bestimmten Subjektposition bei, die von Publika angeeignet werden und weiterführende Subjektivierungsprozesse anleiten kann. Bereits das den Kanälen zugrundeliegende Konzept – die öffentliche Inszenierung von Alltagspraxis durch religiöse muslimische Frauen – verweist dabei auf ein Modell islamischer Weiblichkeit, das sowohl mit der in westlichen Islam-Diskursen hervorgebrachten Figur der unterdrückten Frau bricht als auch das moralische Ideal einer „schamhaften“ Aufmerksamkeitsvermeidung unterläuft (vgl. Nökel 2002, S. 96). So geben sich beide Produzentinnen in ihren Videos extrovertiert und humorvoll, lachen häufig und interagieren in ungezwungenem Tonfall mit dem Publikum. Zudem legen beide in ihren Auftritten Wert auf ein „gestyltes“ Erscheinungsbild, was sich in einer Gestaltung des Körpers durch Make-Up oder Schmuck sowie in wechselnden und aufeinander abgestimmten Outfits äußert (Abb. 1). Damit schließen sie an konventionalisierte kommunikative Formen an, wie sie für Lifestyle- und Influencer-Produktionen auf YouTube typisch sind (vgl. Döring 2019, S. 6 f).

Auch abseits des körperlichen Auftretens der Produzentinnen enthalten die Videos beider Kanäle Elemente, die eine Anschlussfähigkeit an eine breitere Influencer-Kultur bedingen. Zunächst übernehmen sie typische Themen und Formate, wovon einige Konsumpraktiken ins Zentrum rücken: Zu nennen ist etwa der „Haul“ (das Auspacken eines auswärts getätigten Einkaufs), der Produkttest, das „Unboxing“-Video (das Auspacken eines Pakets) oder das Tutorial. Andere Formate zielen hingegen verstärkt auf die Etablierung einer Beziehung zwischen Produzentin und Publikum auf. Beispiele dafür sind etwa die „Story Time“, in der die Produzentin „private“ Anekdoten preisgibt, oder das „Question&Answer“-Format, in dem vom Publikum gestellte Fragen beantwortet werden. Die Formate kehren in regelmäßigen Abständen in sehr ähnlicher Form wieder, „Serialität und Wiederholung“ als zentrale Merkmale der „Influencer-Dramaturgie“ (Nymoen und Schmitt 2021, S. 63) spielen eine wesentliche Rolle.

Genretypische Elemente finden sich auch in der visuellen Ausgestaltung der Videos. Vielfach dominieren Großaufnahmen des Gesichts und der frontale Blick auf die Zuseher_innen die Einstellungen, wodurch sich der Eindruck einer Adressierung und direkten Ansprache des Publikums verfestigen kann (vgl. Hickethier 2012, S. 65). In anderen Fällen übernimmt die Kamera die Perspektive der Darstellerinnen, wodurch ein „authentischer“ und unvermittelter Blick auf ihre materielle Umgebung inszeniert wird. Auffällig ist zudem die genaue und überdeutliche Ausleuchtung der Bilder, die den Videos grundsätzlich einen hellen Charakter verleihen: Wie Hickethier (ebd., S. 78) anmerkt, handelt es sich dabei um eine kulturell konventionalisierte filmische Technik, die eine freundliche Grundstimmung, Glück und Problemlosigkeit signalisieren soll. Neben augenscheinlichen Parallelen in der Inszenierung sind allerdings auch Unterschiede im Hinblick auf die einfließenden Weiblichkeitspositionen zu erkennen, die ich in Folge genauer analysiere.

### Traditionelle Weiblichkeit und moralische Selbstpraxis: „Eurasian Muslima“ als muslimische Mommy-Influencerin

Der erste betrachtete Kanal wird von Nataya betrieben, einer etwa 30-jährigen Schweizerin mit einem thailändischem Elternteil, die nach eigenen Angaben in Folge eines längeren religiösen Suchprozesses zum Islam sunnitischer Prägung konvertierte, mit dem sie durch ihren marokkanisch-stämmigen späteren Ehemann in Kontakt kam (EMA). In einem längeren Video mit dem Titel „Mein Weg zum Islam.“ beschreibt sie den Prozess als eine durch Allah gelenkte Selbsttransformation, wobei sie den Einfluss islamischer YouTube-Prediger explizit hervorhebt (ebd.). Üblicherweise konzentriert sich die mit (teilweise buntem) Kopftuch und in weiten, langen Kleidern auftretende Nataya in ihren Videos aber auf die Darstellung ihres Familienalltags in der ländlichen Schweiz mit anfänglich drei und später vier Kindern, wobei das typische Format längere Vlogs mit bis zu 50 Minuten Dauer sind, in denen das Publikum vor allem Situationen im privaten Rahmen, aber auch Einkäufe oder Familienausflüge über einen oder mehrere Tage hinweg begleitet. Sequenzen, in denen Nataya beim Vollzug von Alltagsaktivitäten zu sehen ist, wechseln sich mit solchen ab, in denen sie sich direkt an Zuseher_innen wendet, diese persönlich anspricht und zu Interaktionen anregt. Ein solcher partizipatorischer Charakter wird dadurch verstärkt, dass Vorschläge des Publikums in Videos berücksichtigt oder Fragen beantwortet werden. Eingestreut sind dabei vermeintlich „authentische“, auf eigenen Erfahrungen basierende Empfehlungen für Produkte, die im Zuge der Alltagspraxis zum Einsatz kommen, wobei das Spektrum von Lebensmitteln und Haushaltsartikeln über Kosmetika bis hin zu religiösen Medien oder Dekorationsobjekten reicht. Ein weiteres zentrales Thema im Erhebungszeitraum stellt Natayas Schwangerschaft dar, der sie mehrere Videos widmet.

**Figure 2:**
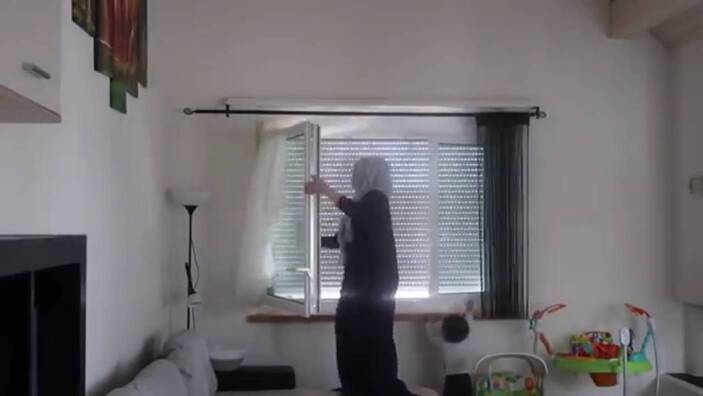

In der klaren Fokussierung der Videos auf Familienleben, Mutterschaft und Haushaltsführung (siehe etwa Abb. 2) tritt zunächst ein traditionelles Modell fürsorglicher und „aufopferungsvoller“ Weiblichkeit hervor, wobei eigene Vorhaben familiären Verpflichtungen untergeordnet werden. Natayas Tagesabläufe erscheinen an den Bedürfnissen der Kinder und des Ehemanns ausgerichtet: In der Auswahl von Kochrezepten merkt sie an, sich an seinem Geschmack zu orientieren (EM16); persönliche Vorhaben – etwa das Färben ihrer Haare oder die Pflege der Fingernägel – werden auf spätere Zeitpunkte aufgeschoben, wie sie in mehreren Fällen berichtet (EM21, EM22). Auch die Inszenierung der partnerschaftlichen Arbeitsteilung rekurriert auf ein traditionelles Modell: Der berufstätige Ehemann, der selten vor der Kamera auftritt, fungiert als „Breadwinner“, wohingegen Erziehungs- und Haushaltstätigkeiten nahezu ausschließlich durch Nataya, die seit der Geburt ihrer Kinder aus dem Erwerbsleben ausgeschieden ist, verrichtet werden. Dabei ist zu berücksichtigen, dass Natayas Tätigkeit als Vloggerin bis zu einem gewissen Grad kommerziell orientiert zu sein scheint, wie Produktempfehlungen und Werbeeinschaltungen nahelegen: Die Inszenierung von Praktiken und Affekten traditioneller Weiblichkeit kann so auch als Strategie von Selbst-Unternehmertum und der Erschließung eines Marktes muslimischer Mütter gelesen werden. Damit kann sie einem Subgenre zugeordnet werden, das als „Family-“ oder „Mommy-Influencing“ bezeichnet wird (vgl. Abidin 2017).

Ein weiteres zentrales Element der Produktionen von *Eurasian Muslima* sind die religiösen Referenzen und Reflexionen, die alle Videos durchziehen: Vor allem findet eine dauerhafte „Sakralisierung“ der Alltagspraxis statt (vgl. Amir-Moazami 2007, S. 184), die sich etwa in der Verwendung religiöser Lob‑, Bitt- und Dankesformeln (etwa Mashallah, Alhamdulillah etc.), der Bitte um Gebete oder dem Verweis auf religiöse Feste oder Verpflichtungen äußert. Auch im Umgang mit den Kindern stellen Islam und islamische Erziehung ein wiederkehrendes Thema dar: So werden sie dazu aufgefordert, islamische Formeln zu wiederholen (EM6), Nataya führt „Quizze“ zu religiösem Wissen durch (EM19), übt mit ihrem ältesten Sohn Koranrezitationen (EM22) oder liest aus islamischen Kinderbüchern vor, die sie wiederum in eigenen Videos rezensiert (EM9). Die Potenziale von alltäglichen Praktiken der Haushaltsführung oder der Care-Arbeit für die Kultivierung islamischer Subjektivität werden klar hervorgehoben.

Darüber hinaus thematisiert Nataya religiöse Fragen auch explizit in ihren Videos, wobei ihre Biografie einen zentralen Bezugspunkt für religiöse (Selbst‑)Reflexionen darstellt. Zentral dabei ist eine Darstellung von islamischer Frömmigkeit und religiöser Alltagspraxis als etwas in einem fortlaufenden Prozess zu Lernendes und zu Trainierendes, wie etwa in folgender Aussage deutlich wird, mit der sich Nataya direkt an ihr Publikum wendet:

Nichts wird einem geschenkt, auch die Beziehung zu Allah Subhanahu wa ta’ala [arabische Lobpreisungsformel, deutsch „er ist gepriesen und erhaben“] nicht. Das ist auch etwas wo man, wo man halt immer dran arbeiten muss. Möge Allah Subhanahu wa ta’ala uns alle in unserem Iman [Glauben] festigen. (EM19)

Wie Nataya in einem anderen Video (EM4) ausführt, reicht religiöses Wissen alleine nicht aus, um den Islam richtig zu leben, vielmehr müssten islamische Themen und Verpflichtungen „gefühlt“ werden: Ein solches Gefühl könne sich durch religiöse Praxis, etwa Gebete, Fasten oder Verschleierung, einstellen, wohingegen der Shaytan [Teufel] auf individueller Ebene eine emotionale Ausrichtung zu bekämpfen versuche, indem er Zweifel nähre und Versuchungen schaffe. In diesen Äußerungen treten Elemente islamisch-moralischer Weiblichkeitspositionen zutage, wie sie etwa von Mahmood (2005) im Kontext der Islamic-Revival-Bewegung beschrieben wurden: Demnach verinnerlichen sich moralisch-religiöse Selbstkonzeptionen mittels konstanter ethischer Selbstpraxis. Die Kultivierung islamischer Frömmigkeit kann demnach nur durch konsequente Übung erfolgen, in der sich religiöse Dispositionen und Emotionen fortlaufend weiterentwickeln und verfestigen (vgl. ebd. S. 135 f).

**Figure 3:**
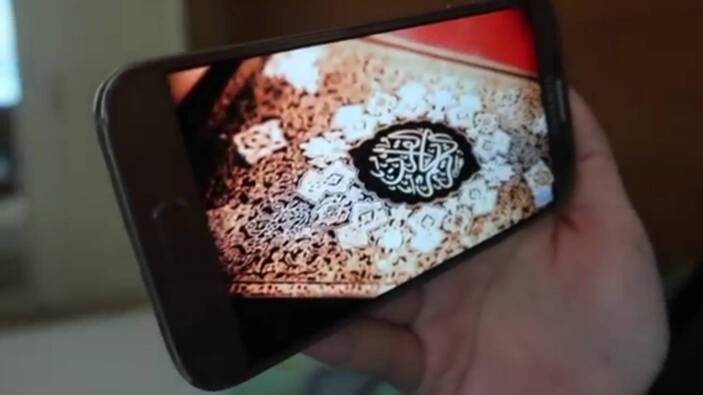

Ergänzt wird ein solcher Wille zur islamischen Selbstformung in Natayas Fall durch den pädagogischen Anspruch, über die Vermittlung von (theoretischem und praktischem) Wissen zu religiösen Selbstbildungsprozessen der Zuseher_innen beizutragen (z. B. in EM4, EM14, EM19, EM22). Entsprechend lässt sie sich als religiöse Influencerin charakterisieren (vgl. Beta 2019, S. 2143). So spricht Nataya Empfehlungen für religiöse Literatur und Internet-Quellen aus (Abb. 3) oder gibt Ratschläge für muslimische Gebets- oder Kleidungspraxis (EM1, EM21, EM22). In diesem Zusammenhang nimmt sie auch auf Friktionen in der Ausübung muslimischer Praxis Bezug, die aus Druck von Seiten des nicht-muslimischen Umfelds (EM4, EM5) oder Kritik innerhalb des islamischen Feldes resultieren:

Für die einen bin ich nicht religiös genug, für die anderen übertreibe ich es total. Ich versuche für mich ein gutes Mittelmaß zu finden. (EM19)

**Figure 4:**
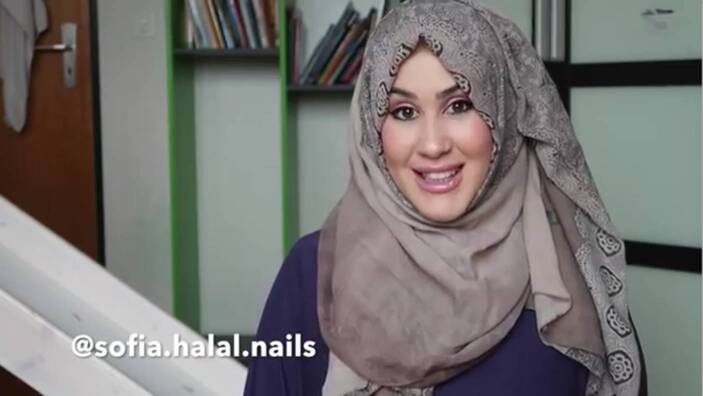

Wie sie selbst anmerkt, speist sich entsprechende Kritik vielfach aus der durch streng religiöse Akteure als „unislamisch“ wahrgenommenen und in Kommentaren oder Privatnachrichten negativ bewerteten Inszenierung ihres Körpers. Üblicherweise trägt sie Make-up, teilweise auch künstliche Wimpern sowie ein Nasen-Piercing, wobei sie in ihren Videos die verwendeten Beauty- und Styling-Produkte rezensiert (EM7, EM11) und ihre Einkäufe dokumentiert (EM7, EM15). Zum Teil betont sie dabei auch den Halal-Status der Produkte, also ihre Zulässigkeit aus islamischer Sicht (Abb. 4). Die Kritik an ihrem Handeln erkennt sie zwar als religiös legitimiert an, betont aber die Unmöglichkeit, alle Regeln zu befolgen, sowie die Priorität des Kopftuchs als Symbol moralischer Weiblichkeit, das andere Vorgaben weniger bedeutsam erscheinen lässt:

Es gibt kein alles oder nichts. Man soll sich auch nicht beeinflussen lassen. […] Die Menschen erkennen [durch das Kopftuch], dass man eine Muslima ist. Geschminkt oder nicht geschminkt. Bunter Hijab oder nicht bunter Hijab. (EM4)

Das Kopftuch wird in ihrem Diskurs somit als Praxis gerahmt, die Handlungsspielräume für die Ausübung anderer weiblicher Selbstpraktiken eröffnet, die aus streng muslimischer Perspektive potenziell kritikwürdig sind. Damit bringt sie ein Modell von muslimischer Weiblichkeit hervor, in dem der Widerspruch zwischen der Kultivierung religiöser Tugenden einerseits und ästhetischer Selbstinszenierung im Sinne bestimmter Schönheitsdiskurse andererseits minimiert wird. Auch im Rahmen eines akzentuiert religiösen Lebens ist eine Formung des Körpers entlang westlich markierter Schönheitskriterien in einem gewissen Rahmen möglich und akzeptabel, so eine zentrale implizite Aussage der Produktionen.

Die verknüpften Subjektivierungspraktiken und die damit in Verbindung stehenden Objekte, Personen und Konzepte werden dabei primär mit positiver Emotion in Verbindung gebracht und als Träger intrinsischen ästhetischen Wertes gerahmt: Der Islam ist *schön* und *perfekt*, das Kopftuch konstituiert die Trägerin als *kostbaren* Schatz *und* sieht gut aus, der Familienalltag macht Spaß und ist harmonisch, die Kinder sind *süß* und *brav*, Nataya *liebt* die gekauften Produkte etc. Elemente islamischer Praxis werden so mit „happy objects“ (Ahmed 2010) des Familienlebens und (vorwiegend alltäglichen) Konsumprodukten zu einem positiv affektiven Arrangement verknüpft und als Konstituenten eines guten muslimischen Lebens hervorgebracht, das sich rund um Ideale von körperlicher Selbstinszenierung, religiöser Selbstkultivierung und traditionellem Familienleben gruppiert. Deutlich tritt eine solche Verhaftung an ein bestimmtes Modellsubjekt zutage, als sie sich nach der Geburt ihres vierten Kindes in einer emotionalen Botschaft aus dem Krankenhaus an ihr Publikum wendet und dazu aufruft, den Wert und die Schönheit alltäglicher Care-Praktiken zu schätzen, die hier als „Privileg“ gerahmt werden:

Die Kinder zu Bett zu bringen ist eine stressige Situation […] man schätzt den Wert irgendwie nicht so. Und wenn man dann plötzlich weg ist, dann lernt man das halt plötzlich ganz anders zu schätzen. […] Alhamdullilah, ich bin so dankbar, dass ich einfach das Privileg habe, bei meinen Kindern zu sein und mich um die zu kümmern. […] Das alleine ist es schon wert, dass man wirklich dankbar sein sollte, zu Allah Subhanahu wa ta’ala, was er uns in unserem Leben geschenkt hat. Diese Baraka [Segen], dass man die Kinder hat [.] (EM18)

Doch auch negative Affekte wie Scham, Wut, Unsicherheit oder Niedergeschlagenheit im Zusammenhang mit Sinnkrisen oder Überforderung mit religiösen und familiären Pflichten werden explizit zum Thema gemacht (EM1, EM4, EM5, EM21, EM22). Vielfach verweisen diese Darstellungen auf strukturelle Momente geschlechtlicher und religiöser Diskriminierung – etwa auf die Ungleichverteilung von Care-Arbeit in familiären Settings, auf die ständige Beobachtung und Bewertung muslimisch-weiblicher Performanzen durch Muslime und Nicht-Muslime oder auf islamfeindliche Tendenzen der Dominanzgesellschaft –, woraus allerdings keine Kritik der zugrundeliegenden Machtverhältnisse resultiert: Vielmehr wird eine Zuwendung zur „Schönheit“ des Islams, Gottvertrauen, Geduld, Dankbarkeit und konsequentere religiöse Selbstformung – etwa durch ein Mehr an Gebet – als Strategie vorgeschlagen, um negative Affekte zu neutralisieren (EM1, EM4).

### Autonomie, Selbst-Unternehmertum und Muslim Fashion: Kubraxdeniz als „Hijabista“-Influencing

Kübra Deniz, die Betreiberin des zweiten Kanals, ist nach eigenen Angaben Social-Media-Influencerin, Studentin der Wirtschaftspsychologie und zusätzlich Mitarbeiterin in einem Unternehmen, lebt in Berlin und hat türkische Wurzeln (KXD12). Sie ist 1993 geboren, trägt Kopftuch (vorwiegend im so genannten Turban-Stil) und ist verheiratet mit dem Fußball-Profi Tunay Deniz. Alltags- und Beziehungsleben steht im Zentrum der etwa wöchentlich erscheinenden und üblicherweise zwischen 10 und 20 Minuten dauernden Videos, die sich ebenfalls als Vlogs einordnen lassen und die Abläufe des Paares – vorwiegend die von Kübra – meist über einen Tag hinweg begleiten. Die Einbindung des Publikums ist auch hier zentral und erfolgt über persönliche Ansprachen, dem Aufruf zu Interaktionen oder die Beantwortung von Publikumsfragen im Rahmen der Videos.

Inhaltlich stehen Konsumpraktiken klar im Vordergrund der Produktionen: Die Darstellung von Einkäufen, Produkttests oder Restaurantbesuchen nimmt einen großen Teil der Zeit in Anspruch. In vielen Fällen werden klare Kaufempfehlungen ausgesprochen, wodurch der Eindruck eines überwiegend kommerziell ausgerichteten Influencer-Kanals vermittelt wird. Die Darstellung von Praktiken ästhetischer Selbstinszenierung im Stil des Hijabista-Trends ist häufig: So enthalten einige Videos Schmink- und Kopftuch-Tutorials, Rezensionen von Kleidungsstücken oder Outfit-Tipps, wobei Kübra mit den Zuseher_innen spricht und die einzelnen Schritte detailliert erklärt. Darüber hinaus sind Treffen mit Freund_innen oder gegenseitige Besuche, gemeinsame Aktivitäten zwischen Kübra und Tunay im privaten Rahmen (beispielsweise gemeinsames Kochen oder Gespräche und Schäkereien), das Gestalten und Dekorieren der Wohnung oder Tunays sportliche Tätigkeit wiederkehrende Elemente in den Videos. Auch Urlaube werden ausführlich in vier Vlogs dokumentiert. Insgesamt wird der Alltag der beiden in den Videos als abwechslungsreich inszeniert, woraus sich das idealisiert erscheinende Bild eines erlebnis- und konsumorientierten urbanen Lebensstils innerhalb eines türkisch-migrantischen Milieus ergibt.

**Figure 5:**
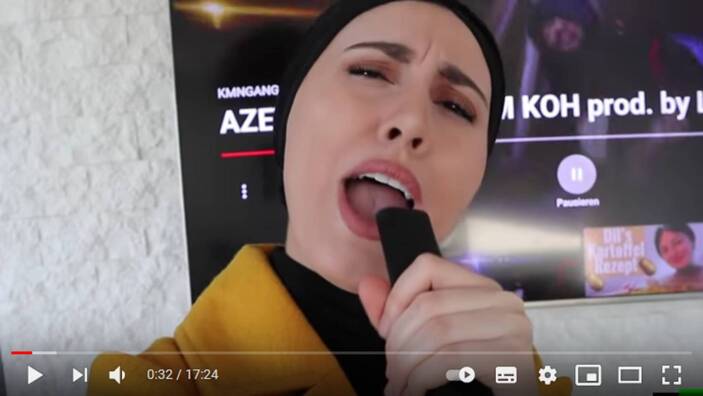

Auch wenn Kübra im Hinblick auf die in den Videos inszenierten Alltagspraktiken (Shopping, Beauty etc.) dominante Zuschreibungen an Weiblichkeit reproduziert, bricht sie an anderer Stelle damit: So verweist sie mehrfach auf ihre Vorliebe für Rap-Musik (KXD1, KXD5, KXD13, siehe Abb. 5) oder verwendet in ihrem Sprechen (teils männlich kodierte) Kraftausdrücke, die ein moralisches Ideal zurückhaltender oder schamhafter Weiblichkeit konterkarieren, wie etwa in folgendem Zitat deutlich wird:

Ich werd mich nicht soooo krass schminken. Das sag ich immer und dann hab ich so fünf Tonnen Make-Up auf der Fresse. (KXD12)

**Figure 6:**
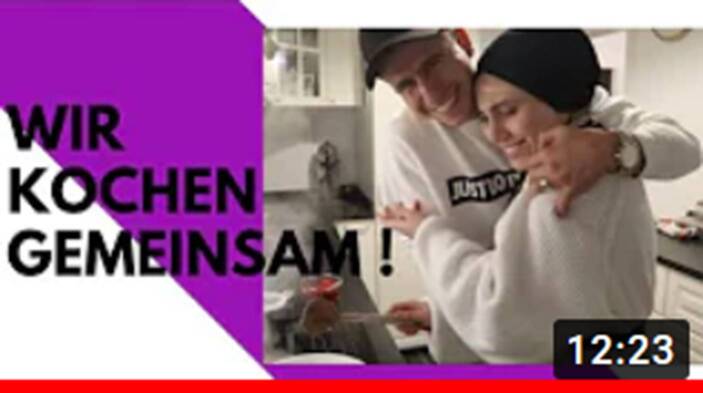

Im Hinblick auf partnerschaftliche Ideale findet eine Abgrenzung zum traditionellen Modell, wie es im Fall von *Eurasian Muslimah* hervortritt, statt. Zwar wird deutlich, dass legitimes Beziehungsleben eine Eheschließung voraussetzt, die Partnerschaft selbst wird allerdings als grundsätzlich egalitäres Unterfangen inszeniert: Haushaltsarbeiten werden im Rahmen der Videos vielfach gemeinsam durchgeführt (siehe auch Abb. 6), wesentliche Entscheidungen in der Zukunftsplanung gemeinsam getroffen und im Fall von Interessenskonflikten Kompromisse ausgehandelt: Das wird etwa deutlich, wenn Kübra berichtet, dass das Paar trotz Tunays Wechsel zu einem auswärtigen Fußballverein seine Wohnung in Berlin behalten wird, damit Kübra nicht ihrem sozialen Umfeld entrissen wird (KXD25). Expliziert wird dieses Ideal auch in einem Video, in dem die beiden von ihren Hochzeitsvorbereitungen berichten:

Versucht ansonsten immer die Mitte zu finden […] Versucht einfach das […] zu machen, was ihr beide wollt, und versucht nicht, bei allem eure Eltern miteinzubeziehen. Das macht auch einiges kaputt. (KXD22)

In diesem Zusammenhang lassen sich wiederholt Fragmente eines diskursiven Ideals weiblicher Unabhängigkeit erkennen: So ruft Kübra etwa ihre Zuseherinnen auf, „Männern nicht hinterherzurennen“ (KXD22), kritisiert gewisse Formen der elterlichen Einmischung in Eheangelegenheiten der Kinder als „hinterwäldlerisch“ (ebd.) oder weist nach einer entsprechenden Publikumsfrage darauf hin, dass eigene Kinder für sie derzeit noch kein Thema seien (KXD12). Des Weiteren inszenieren die Videos häufig Situationen, in denen Kübra Tunay widerspricht und eigene Wünsche und Präferenzen klar zum Ausdruck bringt. Auch wenn es dabei zumeist um ästhetische Fragen (etwa im Hinblick auf Bekleidung oder Wohnungsdekoration, KXD3) geht, lassen sich hierin Elemente einer Performance selbstbestimmter und selbstbewusst auftretender Weiblichkeit erkennen.

**Figure 7:**
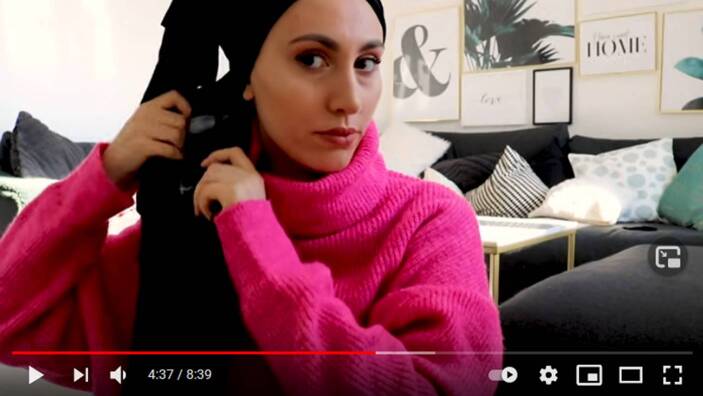

Im Vergleich zu *Eurasian Muslima* wird religiöse Praxis in den Produktionen von *kubraxdeniz* nur in Einzelfällen explizit angesprochen, etwa wenn anlässlich der Geburt eines Kindes von Freund_innen auf muslimische Rituale Bezug genommen (KXD10), das Freitagsgebet durchgeführt (KXD13, KXD15) oder das gemeinsame Fasten im Zuge des Ramadans dokumentiert (KXD28) wird. Zudem sind Bitt‑, Dankes- und Lobformeln wie „bismillah“ oder „mashallah“ in einigen Videos präsent. Angesichts des geringen Ausmaßes an expliziter religiöser Reflexion wäre es naheliegend, von einer Art „ungebundener Restreligiösität“ (Aslan et al. 2017) auszugehen. Dagegen spricht allerdings die Bekleidungspraxis von Kübra, die, wie an mehreren Stellen deutlich wird, durchaus von einem moralisch-religiös geformten Diskurs weiblicher Sittsamkeit informiert ist: So erklärt sie in einem Video, dass sie Wert auf die Bedeckung ihres Halses und sämtlicher Haare lege. Um eine solche „korrekte“ Verschleierung mit modischen Ansprüchen zu versöhnen, verfolgt sie Strategien wie etwa das Tragen weiter Rollkragen- oder Kapuzenpullover (siehe auch Abb. 7):

Ich trag dann immer Rollkragenpullis, damit man nicht allzu sehr meinen Hals sieht und darunter trag ich ein Bone, nennt sich das, quasi etwas, das die kompletten Haare so zusammenhält. Meine Babyhärchen kommen deswegen auch nicht raus oder so. (KXD22)

Auch im Urlaub auf den Malediven trägt Kübra ausschließlich langärmlige Kleidung, die das Gesäß bedeckt (KXD15). Ebenso ist es ihr wichtig, dass ihre Kleidung nicht transparent und darunter keine Haut sichtbar ist (KXD25). Wenn auch in gegenläufiger Gewichtung, fließen in die von Kübra performativ hervorgebrachte Subjektposition Fragmente islamischer Moraldiskurse einerseits und populärkultureller Schönheits- und Lifestyle-Diskurse andererseits ein, wobei letzteren eine höhere Sichtbarkeit zugestanden wird. Auch im Rahmen einer akzentuiert ästhetischen Stilisierung des Körpers kann islamische Selbstkultivierung stattfinden, so die Aussage, die jene von *Eurasian Muslima* gewissermaßen invertiert.

**Figure 8:**
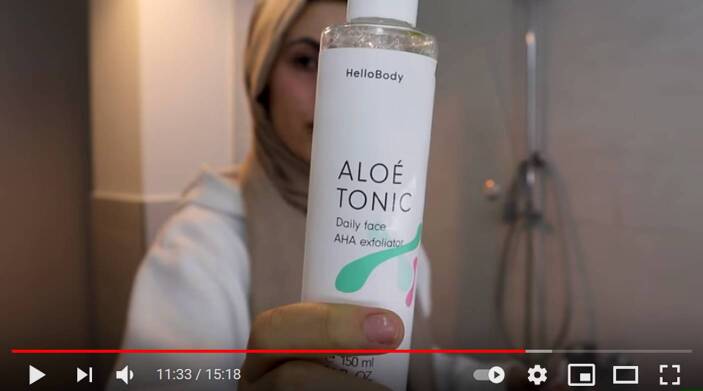

Deutlicher tritt im Fall von *kubraxdeniz* die kommerzielle Orientierung des Kanals in Erscheinung: Klare Produktwerbung, zumeist in Form „persönlicher“ Empfehlungen und Kooperationen mit Werbepartnern, insbesondere im Bereich von Beauty- und Wellness-Produkten, ist in nahezu allen Videos präsent (Abb. 8). So entsteht der Eindruck, dass Fragmente eines Diskurses weiblicher Sittsamkeit hier im neoliberalen Diskurs eines „unternehmerischen Selbst“ (Bröckling 2005, 2007) aufgehen, wobei die gezielte Mobilisierung von Praktiken und Symbolen ethischer Weiblichkeit für kommerzielle Zwecke als Strategie für islamisch-weibliche Partizipation im Geschäftsleben interpretiert werden kann. Kübra inszeniert sich als aktive, unabhängige und erfolgreiche muslimische Frau, die neben dem Studium und ihrer Tätigkeit in einem Unternehmen an ihrer Influencer-Karriere arbeitet. Das wird etwa an ihrer Reaktion deutlich, als sie in den YouTube-Kommentaren für ihre eingeschränkten häuslichen Fähigkeiten (bzw. ihre Mutter für ihre unzulängliche Erziehung) kritisiert wird:

Meine Mutter hat mir beigebracht das [sic] SCHULE, UNIVERSITÄT und EMANZIPATION wichtig im Leben sind, nicht das KOCHEN KÖNNEN!!! Und wegen der Erziehung meiner MUTTER, bin ich gerade PSYCHOLOGIN, CEO & MANAGER in einem Top Unternehmen. (KXD2)

Worauf Kübra Bezug nimmt, wenn sie sich in diesem vom Publikum mit mehreren Hundert „Upvotes“ gewürdigten Kommentar als „CEO & MANAGER“ bezeichnet, ist zwar nicht ganz klar, deutlich tritt aber ein Ideal hervor, das eine Verquickung von Emanzipation und wirtschaftlichem Erfolg als zentrales Element kompetenter Weiblichkeit in den Vordergrund rückt und somit die „Werte der neuen Meritokratie“ (McRobbie 2010, S. 92) repräsentiert. Auch ihre Follower_innen werden aufgerufen, diesem Beispiel zu folgen und ihre ästhetischen Selbstpraktiken zu kommodifizieren, wenn etwa eine App beworben wird, auf der Selfies hochgeladen und durch die Verlinkung von Kleidungsstücken Einnahmen erzielt werden können:

Ihr seid sowieso zuhause und macht sowieso Outfitbilder oder Spiegelselfies, ihr macht also mit Nichtstun Geld. Was gibt’s Besseres? (KXD17)

Säkulare Ideale weiblicher Autonomie und körperlicher Selbstästhetisierung werden also gemeinsam mit religiösen Vorstellungen weiblicher Moralität in eine ökonomische Logik eingespeist, woraus das Modell eines „lustvollen“ Lebens resultiert, das vor allem in affektiv aufgeladenen Konsum- oder Interaktionspraktiken hervorgebracht wird: Diese werden teilweise mit der Darstellung intensiver Erregungszustände verknüpft (etwa einem freudigen Schreien); auch der Umgang mit anderen Personen, etwa Tunay oder Freundinnen, wird vielfach von einer Inszenierung emotionaler Intensität begleitet. Im Gegensatz zu Eurasian Muslima spannt Kübra affektive Relationen zudem über eine stärker jugendkulturell geprägte Terminologie auf, was deutlich wird, wenn gewisse Objekte, Praktiken oder Personen etwa als *krass, cool, geil* oder *fresh* klassifiziert werden. Negative Affekte und Spannungsverhältnisse in der Lebenswelt junger Muslim_innen werden in den Videos hingegen nur am Rande behandelt, wenn etwa auf Drucksituationen in familiären Konstellationen Bezug genommen wird. Das mag zwar im unterhaltenden und kommerziellen Anspruch der Videos begründet liegen, weist aber insofern eine ideologische Komponente auf, als dass das hierbei hervorgebrachte Modell eines spaßbetonten und erfolgreichen muslimischen Lebens für viele junge Muslim_innen aus verschiedenen Gründen nicht zu realisieren ist: Sozioökonomische Marginalisierung (vgl. Geier und Gaupp 2015), Diskriminierungen im Berufsleben und der Öffentlichkeit (vgl. Sauer 2007) oder patriarchale Familienstrukturen (vgl. Wensierski 2014, S. 315ff) stellen Faktoren dar, die Handlungsmöglichkeiten junger Muslim_innen systematisch begrenzen und eine Angleichung an das von Kübra hervorgebrachte Ideal erschweren.

## Fazit

Trotz der augenscheinlichen Unterschiede der Kanäle, die sich insbesondere in der Explizitheit der Thematisierung religiöser Praktiken und dem Ausmaß an offen kommerziellen Inhalten äußert, möchte ich vorschlagen, dass beide einer übergreifenden diskursiven Formation zugeordnet werden können, die ein spezifisches Modell gelingenden und erfüllenden islamischen Lebens im westlichen Kontext zeichnet und eine damit verbundene Form islamischer Subjektivität über Social-Media-Formate hervorbringt: Zentral für diese *ethisch/ästhetisch-lifestyle-orientierte* Subjektposition ist die performative Auflösung von Widersprüchen zwischen säkularer Konsum- und Populärkultur einerseits sowie islamischer Religiosität andererseits in einem Hybridentwurf, der auf einer „produktiven Liminalität“ (Elberfeld und Otto 2009, S. 11) von ethischer und ästhetischer Selbstpraxis basiert. So entsteht ein erweitertes Modell davon, was im Rahmen eines islamischen Lebensstils als zulässig betrachtet werden kann, wobei konkrete Räume des aus muslimischer Perspektive legitim Mach‑, Sag- und Sichtbaren eröffnet werden. Von früheren Formen eines hybriden Neo-Islams grenzt sich der von mir beschriebene Subjekttypus insbesondere durch den Fokus auf Konsumpraktiken und die kommerziell orientierte, selbst-vermarktende Komponente ab, die als mit religiöser Praxis vereinbar dargestellt wird.

In Anlehnung an Foucault (1994) lassen sich Körper und Affekte als die *ethisch/ästhetische* Substanz dieses Subjektmodells begreifen, die anhand von vergeschlechtlichten Moral- und Schönheits-Diskursen zu formen ist und somit sowohl göttlichen als auch profanen *Instanzen* unterworfen wird. Ebenso oszillieren die damit verbundenen *Techniken der Selbstbearbeitung* zwischen genuin religiöser und nicht-religiöser Praxis, wobei letztere auch immer von ethischen Gehalten durchdrungen ist und nicht gänzlich unabhängig von religiösen Wissensformen gedacht werden kann, wie das wiederkehrende Thema des Kopftuch-Styles zeigt. Umgekehrt muss auch die explizit religiöse Praxis als doppelt ästhetisch betrachtet werden: einerseits im Sinne einer religiösen Ästhetik, die moralische Modelle als begehrenswerte Träger ästhetischer Werte konstituiert; andererseits im Sinne einer säkularen Ästhetik, die eine Zurichtung weiblicher Körper im Sinne bestimmter Schönheitsdiskurse fordert. Wichtig ist dabei zudem die Rahmung der Praktiken als freiwillige Akte sowie ihre Inszenierung als Ausgangspunkt positiver Affizierung. Als *Telos* eines solchen ethisch-ästhetischen Projekt der Selbstkultivierung, so möchte ich vorschlagen, wird folglich ein gutes Leben ausgegeben, in dem die Widersprüche diskursiver Referenzsysteme neutralisiert werden und eine Aussöhnung konkurrierender Anforderungsprofile in einem positiv affizierenden Modellsubjekt stattfindet.

Wie ich gezeigt habe, schaffen die Kanäle und Videos eine hybride Subjektposition, die versucht, praktische Modelle zur Überwindung von Spannungsverhältnissen zwischen den divergierenden Weiblichkeitsdiskursen, die in den Alltagswelten junger Musliminnen Widerhall finden, anzubieten. Die entsprechenden Ambivalenzen und Probleme werden nur zum Teil thematisiert – im Fall von *Eurasian Muslima* etwas ausführlicher – und noch seltener kritisiert. Der Fokus liegt vielmehr auf der Vermittlung und Vermarktung positiver Entwürfe hybrider Weiblichkeit, die ethisch-religiöse mit modisch-expressiven und neoliberal selbst-unternehmerisch geprägten Anforderungsprofilen aussöhnt (vgl. dazu auch Peterson 2017, S. 17 f). Damit lassen sich die Produktionen als Elemente einer zeitgenössischen muslimischen Jugendkultur im Westen begreifen, die im „Dialog“ mit breiteren populärkulturellen Formaten und Diskursen Widersprüche zwischen konkurrierenden religiösen und säkularen Selbstentwürfen aufzulösen suchen.

## Supplementary Information




## Footnotes

1 Unter Jugendlichen in Deutschland stellt YouTube das beliebteste und meistgenutzte digitale Medium dar, wie eine repräsentative Studie für das Jahr 2019 belegt (vgl. mpfs 2020).

2 Spreche ich hier von Diskurs, beziehe ich mich auf einen von Michel Foucault (2015) geprägten Diskursbegriff, der die Wissensproduktion durch zeichenförmige Aussagepraktiken akzentuiert. Ein Diskurs kann demnach als abstraktes, historisch verortetes und in Machtkonstellationen eingebundenes System von Aussagen begriffen werden, die ihre Gegenstände regelgeleitet zueinander in Beziehung setzen und somit in sozial verstehbarer Form hervorbringen (vgl. ebd. S. 70–73; Keller 2013, S. 30).

3 Im Anschluss an theoretische Positionen der *Affect Studies* soll Affekt hier grundsätzlich als dauerhafter und in konkreten Begegnungen hervortretender Strom von Intensitäten zwischen menschlichen oder nicht-menschlichen Körpern begriffen werden (vgl. Seyfert 2012, S. 30 f), wohingegen Emotionen sozial konventionalisierte Artikulationen bestimmter Affizierungsdynamiken darstellen.

4 Die Assoziation des Kopftuchs mit weiblicher Unterdrückung hat eine lange Geschichte und stellt ein wiederkehrendes Thema in der westlichen Auseinandersetzung mit dem Islam dar, wobei es vielfach als Indiz islamischer Rückständigkeit gedeutet und zur Legitimationsgrundlage für Herrschaftsansprüche gegenüber dem „Orient“ wurde (vgl. Ahmed 1992, S. 150ff).

5 Hierbei wird wiederum deutlich, dass – entgegen homogenisierender Tendenzen des hegemonialen Islam-Diskurses – *der* Islam als religiöse Gruppe nicht existiert, sondern ein breites Spektrum an Strömungen, Konfessionen, Schulen, Organisationen, geographisch-kulturellen Traditionen, Alltagspraktiken und letztlich auch Weiblichkeitspositionen umfasst (vgl. dazu auch Reddig 2011, S. 159 f).
