# Supplementary material for: Piety, fashion and business: positions of ethical/aesthetic femininity in muslim lifestyle-vlogs
Source: Z Relig Ges Polit. 2021 Sep 3;5(1):243–70. [Article in German] doi: 10.1007/s41682-021-00068-y (PMC8700261; doi:10.1007/s41682-021-00068-y)
Supplement: Supplementary file 1 [file 41682_2021_68_MOESM1_ESM.docx]

**Appendix: Videos der analysierten Kanäle**

1. **Eurasian Muslima**

| Code | Name | Datum | Link |
| --- | --- | --- | --- |
| EMA | Mein Weg zum Islam. | 24.04.2018 | <https://youtu.be/MgooRsQa6bk>  Zugegriffen 30.12.2020 |
| EM1 | VLOG \| URLAUB IN DEN BERGEN \| BELEIDIGT IM EINKAUFSZENTRUM \| IS RÜCKKEHRERINNEN \| KANALEMPFEHLUNGEN | 15.07.2018 | <https://youtu.be/NGxXmnmS5Qo>  Zugegriffen 30.12.2020 |
| EM2 | ES GIBT NEUIGKEITEN...🙈UPDATE! | 22.08.2018 | <https://youtu.be/-MIHeBL-J8A>  Zugegriffen 30.12.2020 |
| EM3 | 6-12SSW \| UPDATE \| ERSTES TRIMESTER BABY#4 \| SYMPTOME UND WIE WIR ES HERAUSGEFUNDEN HABEN ❤ | 24.08.2018 | <https://youtu.be/sg_sWLOrUS8>  Zugegriffen 30.12.2020 |
| EM4 | WARUM ICH DEN HIJAB 5 MAL ABGELEGT HABE \| MEINE HIJAB STORY. | 29.08.2018 | <https://youtu.be/ASVwkSL--MA>  Zugegriffen 30.12.2020 |
| EM5 | ZUM ISLAM KONVERTIERT \| SO HABE ICH ES MEINEN ELTERN GESAGT | 03.09.2018 | <https://youtu.be/Tzzo-ZaRCDc>  Zugegriffen 30.12.2020 |
| EM6 | YOUNES LÄUFT!!!😍 \|\|ULTRASCHALLVIDEO, GESCHLECHT DES BABYS?! \| FAMILIENVLOG | 07.09.2018 | <https://youtu.be/3njyemGyoF0>  Zugegriffen 30.12.2020 |
| EM7 | \| XXXL DM HAUL \| FAMILIENEINKAUF \| | 10.09.2018 | <https://youtu.be/d61lCepcRbM>  Zugegriffen 30.12.2020 |
| EM7a | ARROZ CON POLLO \| ONE POT GERICHT \| HÄHNCHEN-REISPFANNE \| REZEPT | 20.09.2018 | <https://youtu.be/XgITTfy_JoA>  Zugegriffen 10.04.2021 |
| EM8 | 13-15 SSW \| UPDATE \| MEINE GEWICHTSZUNAHME \| ERSTTRIMESTER-TEST | 13.09.2018 | <https://youtu.be/g0crMAT3ES0>  Zugegriffen 30.12.2020 |
| EM9 | ISLAMISCHE KINDERBÜCHER 🕋🕌 \|\| UNSERE EMPFEHLUNGEN | 17.09.2018 | <https://youtu.be/6GGVtd_6hxI>  Zugegriffen 15.04.2020 |
| EM9a | EUROPAPARK VLOG \| ERSTE ACHTERBAHNFAHRT FÜR DIE KINDER \| PANNE AUF EINER BAHN! \| | 24.09.2018 | <https://youtu.be/hFr6hjLQ1aA>  Zugegriffen 10.04.2021 |
| EM9b | 5 TAGE FOOD DIARY \| MEINE ERNÄHRUNG IN DER SCHWANGERSCHAFT | 25.10.2018 | <https://youtu.be/zJi8b6mSqa0>  Zugegriffen 10.04.2021 |
| EM10 | ALLTAG ALS SCHWANGERE 3-FACH MAMA \| FOOD HAUL & LECKERES TORTILLA REZEPT \| FAMILIEN VLOG | 10.11.2018 | <https://youtu.be/wH7T8SlauL8>  Zugegriffen 15.04.2020 |
| EM11 | HALAL NAGELLACK?!? ICH TESTE, IHR ENTSCHEIDET! \| SOFIA HALAL NAILS | 14.11.2018 | <https://youtu.be/UNS4qgjgtos>  Zugegriffen 30.12.2020 |
| EM12 | SCHWANGERSCHAFTS-UPDATE \| 20-25SSW \| 3 TRIMESTER \| IMPFEN IN DER SS?! \| WAS WIRD BABY #4?!😍 | 20.11.2018 | <https://youtu.be/BiY5Ch3Ki_Y>  Zugegriffen 10.04.2021 |
| EM13 | SAMSTAGS VLOG \| KEIN PLATZ FÜR UNS IM SHOPPINGCENTER?! \| ICH WERDE VERWÖHNT😍 \| NEUES FILMLICHT | 16.12.2018 | <https://youtu.be/BiY5Ch3Ki_Y>  Zugegriffen 10.04.2021 |
| EM14 | XXL FAMILIENVLOG \| 1 WOCHE ALS 3-FACH MAMA IM 8. MONAT \| GEBETE INTEGRIEREN, SCHMINKE \| BÜCHER HAUL😍 | 12.01.2019 | <https://youtu.be/gCO7PJBuYms>  Zugegriffen 15.04.2020 |
| EM15 | XXL HALAL FOOD HAUL \| MONATSEINKAUF FÜR EINE 5 KÖPFIGE FAMILIE \| TÜRKISCHER LADEN & ASIASHOP | 08.02.2019 | <https://youtu.be/Mk425AUTGzk>  Zugegriffen 15.04.2020 |
| EM16 | LAMMCURRY REZEPT \|38SSW, ENDSPURT \| BABYCOUNTDOWN😍 IKEA HAUL \| MAMA ALLTAG | 12.02.2019 | <https://youtu.be/N3zQcyLZ-n0>  Zugegriffen 10.04.2021 |
| EM17 | UNSER SONNTAG \| GRILLEN MIT 3 KINDERN \| DAS NEHMEN WIR MIT😋 \| 39SSW, GEHT'S LOS?!?😮 \| FAMILIENVLOG | 19.02.2019 | <https://youtu.be/YQq5wcztYd4>  Zugegriffen 10.04.2021 |
| EM17a | XXL ALDI & LIDL FOOD HAUL \| UNSER FAMILIENEINKAUF MIT 3 KINDERN | 22.02.2019 | <https://youtu.be/Z5l6s3q195c>  Zugegriffen 10.04.2021 |
| EM18 | UNSER 4TES WUNDER IST DA🌺 \| WEHENTAGEBUCH \| BLASENSPRUNG \| GEFÜHLSCHAOS IM KRANKENHAUS \| VLOG | 09.03.2019 | <https://youtu.be/c1UORbQ-7n0>  Zugegriffen 30.12.2020 |
| EM19 | ALLTAG MIT 4 KINDER \| FRÜHLINGSPUTZ \| EURE VERMUTUNGEN ÜBER MICH😱! \| ALTE KINDERFOTOS🙈\|XXL VLOG | 18.04.2019 | <https://youtu.be/QDNGaiXInbg>  Zugegriffen 30.12.2020 |
| EM20 | STURZGEBURT IM WASSER \| Natürliche Wassergeburt ohne Schmerzmittel \| 4tes Kind \| MEIN GEBURTSBERICHT | 23.03.2019 | <https://youtu.be/gN1tK9mYTdA>  Zugegriffen 10.04.2021 |
| EM21 | FAMILIENEINKAUF \| RAMADAN HAUL&REZEPT \| ASIYAH LACHT😍 \| 1 WOCHE ALS 4-FACH MAMA | 04.05.2019 | <https://youtu.be/7511bMgADLo>  Zugegriffen 10.04.2021 |
| EM22 | RAMADAN FAMILIENVLOG \| BRUNNEN NAMEN😍 \| SCHLECHTE MÜTTER?! \| KOCHT HARIRA UND RISOTTO MIT MIR😋 | 11.05.2019 | <https://youtu.be/SiAwM59cwLc>  Zugegriffen 30.12.2020 |

1. **Kubraxdeniz**

| Code | Name | Datum | Link |
| --- | --- | --- | --- |
| KXD1 | KMN LIVE KONZERT BERLIN mit AZET ZUNA ENO CAPITAL BRA & Abschnitte aus unserer Wohnung ! | 08.10.2018 | <https://youtu.be/4pNpEyS896A>  Zugegriffen 30.12.2020 |
| KXD2 | WITZIGSTE JELLY BELLY CHALLENGE EVER ! Mit hamsi kuzu ❤️ | 21.10.2018 | <https://youtu.be/kF7fWJzhtUc>  Zugegriffen 30.12.2020 |
| KXD3 | WOHNUNGS VLOG WESTWING MÖBEL ZU GÜNSTIGEN PREISEN ! IKEA & PRIMARK HOME | 25.10.2018 | <https://youtu.be/bKt4incHGiE>  Zugegriffen 30.12.2020 |
| KXD4 | Dilarakaynarca und Oguzaksy zu Besuch bei uns in BERLIN !\| VLOG 🍔 💄🛍 | 30.10.2018 | <https://youtu.be/kejXE5X8hAk>  Zugegriffen 30.12.2020 |
| KXD5 | Goodbye Dilara & Oguz & Hello New Week WIMPERNLIFTING & BABYKATZEN VLOG KUBRAXDENIZ | 11.11.2018 | <https://youtu.be/4IBUtWfx1bE>  Zugegriffen 30.12.2020 |
| KXD6 | Daily VLOGS ? Bringe ich ein Mashup raus ? KLARTEXT ! \| KUBRAXDENIZ | 18.11.2018 | <https://youtu.be/KSUxxBrpKz0>  Zugegriffen 15.04.2020 |
| KXD7 | Habe ich Depressionen ? Meine Panikattacken & Panikattacken loswerden | 19.11.2018 | <https://youtu.be/1A4CFIp6igA>  Zugegriffen 15.04.2020 |
| KXD8 | WÜRMER in der WOHNUNG ! Was kostet dein Outfit ? 10 TAUSEND Euro ! 😲 | 22.11.2018 | <https://youtu.be/jA4OL-lZPzU>  Zugegriffen 30.12.2020 |
| KXD9 | Verarscht vom ZARA KASSIERER ?! FAKE KASSENBELEG ! Black Friday HAUL 2018 ! | 25.11.2018 | <https://youtu.be/sJwZ3EwiXUk>  Zugegriffen 15.04.2020 |
| KXD10 | ALLTAGSVLOG ! FREUNDE ! UNZENSIERT ! 100% CHAOS !! 😂 | 26.11.2018 | <https://youtu.be/Xpe2n2PFVZQ>  Zugegriffen 30.12.2020 |
| KXD11 | What's in my BAG ?? \| Shopping Fieber 🛍 \| XXL VERLOSUNG ! | 28.11.2018 | <https://youtu.be/HxacJHqgGM4>  Zugegriffen 30.12.2020 |
| KXD12 | WIE VERDIENE ICH MEIN GELD ?! 💰 BLOGGER LEBEN & TURKISH WEDDING 🇹🇷 \| XXL VLOG & Q&A | 03.12.2018 | <https://youtu.be/iIA-EQsa9hM>  Zugegriffen 30.12.2020 |
| KXD13 | 👫 EHE & BEZIEHUNGSPROBLEME STORY TIME & Urlaubsvorbereitungen ! ✈ | 10.12.2018 | <https://youtu.be/75-zYOZgZuQ>  Zugegriffen 30.12.2020 |
| KXD14 | Unsere Reise auf die Malediven ! 10 Stunden Flug & Ankunft 🏖 URLAUB VLOG | 21.12.2018 | <https://youtu.be/VACA3tepJ30>  Zugegriffen 30.12.2020 |
| KXD15 | Wie viel haben wir für die Malediven bezahlt ? 💰 XXL MALEDIVEN VLOG 🌴 \| KUBRAXDENIZ | 01.01.2019 | <https://youtu.be/USDnkMiP5nI>  Zugegriffen 30.12.2020 |
| KXD16 | MAC, ESTEE LAUDER & MICHAEL KORS zu SCHNÄPPCHEN PREISEN ! ISTANBUL VLOG \| KUBRAXDENIZ | 13.01.2019 | <https://youtu.be/V4LM5sTcK-U>  Zugegriffen 30.12.2020 |
| KXD17 | Geld verdienen mit Selfies !! & das LECKERSTE LASAGNE REZEPT auf der Welt !! \| VLOG KUBRAXDENIZ | 20.01.2019 | <https://youtu.be/DreZhr32NB0>  Zugegriffen 30.12.2020 |
| KXD18 | Meine Morgenroutine & ein Riesen Pool nur für mich ! VLOG | 27.01.2019 | <https://youtu.be/USAlqfgUmy4>  Zugegriffen 15.04.2020 |
| KXD19 | 1 Woche Fernbeziehung! XXL Antalya VLOG | 03.02.2019 | <https://youtu.be/UDVd8fl2N9I>  Zugegriffen 30.12.2020 |
| KXD20 | MEIN GEBURTSTAGSGESCHENK! \| Die Reise nach XXX \| kubraxdeniz | 06.02.2019 | <https://youtu.be/3kSuq6MYNHA>  Zugegriffen 30.12.2020 |
| KXD21 | HORROR FLUG! Urlaubsvlog & Roomtour in Budapest \| kubraxdeniz | 16.02.2019 | <https://youtu.be/Dra_e2RJl-A>  Zugegriffen 30.12.2020 |
| KXD22 | EASY TURBAN TUTORIAL & Alltagsvlog \| kubraxdeniz | 20.02.2019 | <https://youtu.be/Ms0YvXrw4QU>  Zugegriffen 30.12.2020 |
| KXD23 | MEIN ANKLEIDEZIMMER \| Wohnungsvlog \| kubraxdeniz | 24.02.2019 | <https://youtu.be/sw7QuGBOXZw>  Zugegriffen 30.12.2020 |
| KXD24 | WIR WOLLTEN HEIRATEN & DURFTEN NICHT ! 😭👰 | 24.03.2019 | <https://youtu.be/ZNXmIsvdVx0>  Zugegriffen 30.12.2020 |
| KXD25 | WIR ZIEHEN UM ! 😭 \| WOHNUNGSDEKORATION trotz Umzug ! \| VLOG kubraxdeniz | 31.03.2019 | <https://youtu.be/1z8bpOjH860>  Zugegriffen 30.12.2020 |
| KXD26 | IKEA BALKON TOUR \| INSPIRATION 😍 KUBRAXDENIZ | 30.04.2019 | <https://youtu.be/Se4-uvwhkFY>  Zugegriffen 30.12.2020 |
| KXD27 | 🇹🇷TURKISH WEDDING GET READY WITH ME 🇹🇷 | 05.05.2019 | <https://youtu.be/5Iq6NkO_Gcs>  Zugegriffen 30.12.2020 |
| KXD28 | EIN TAG RAMADAN... \| FASTEN im Alltag \| KUBRAXDENIZ VLOG | 15.05.2019 | <https://youtu.be/AIoyPuLiL_A>  Zugegriffen 30.12.2020 |
| KXD29 | Mein HOCHZEITSEINKAUF & CEYIZ ! ZARA HOME DUPES ab 1€ ! 😱 | 17.06.2019 | <https://youtu.be/QG1uCoaXsAg>  Zugegriffen 15.04.2020 |
| KXD30 | HOCHZEITSFOTOS DIE ICH NIE GEZEIGT HABE 🙈 Alltagsvlog kubraxdeniz | 30.06.2019 | <https://youtu.be/cUI1R3_5_nI>  Zugegriffen 30.12.2020 |
